# Breakup morphology of expelled respiratory liquid: From the perspective of hydrodynamic instabilities

**DOI:** 10.1063/5.0022858

**Published:** 2020-09-01

**Authors:** M. Vadivukkarasan, K. Dhivyaraja, Mahesh V. Panchagnula

**Affiliations:** 1 Department of Mechanical Engineering, National Institute of Technology Puducherry, Karaikal 609609, India; 2 Department of Applied Mechanics, Indian Institute of Technology Madras, Chennai 600036, India

## Abstract

Understanding the breakup morphology of an expelled respiratory liquid is an emerging interest in diverse fields to enhance the efficacious strategies to attenuate disease transmission. In this paper, we present the possible hydrodynamic instabilities associated with expelling the respiratory liquid by a human. For this purpose, we have performed experiments with a cylindrical soap film and air. The sequence of the chain of events was captured with high-speed imaging. We have identified three mechanisms, namely, Kelvin–Helmholtz (K–H) instability, Rayleigh–Taylor (R–T) instability, and Plateau–Rayleigh (P–R) instability, which are likely to occur in sequence. Furthermore, we discuss the multiple processes responsible for drop fragmentation. The processes such as breakup length, rupture, ligament, and drop formation are documented with a scaling factor. The breakup length scales with *We*^−0.17^, and the number of ligaments scales as $\sqrt{Bo}$. In addition, the thickness of the ligaments scales as *We*^−0.5^. Here, *We* and *Bo* represent the Weber and Bond numbers, respectively. It was also demonstrated that the flapping of the liquid sheet is the result of the K–H mechanism, and the ligaments formed on the edge of the rim appear due to the R–T mechanism, and finally, the hanging drop fragmentation is the result of the P–R instability. Our study highlights that the multiple instabilities play a significant role in determining the size of the droplets while expelling a respiratory liquid. This understanding is crucial to combat disease transmission through droplets.

## I. INTRODUCTION

The novel and respiratory infectious coronavirus disease (COVID-19) has proliferated and spread exponentially across the globe in recent days. Understanding the dynamics of transmission routes is a primary concern among researchers in diverse fields.^1^ A few possible fluid mechanical routes of disease transmission happen via human respiratory activities such as breathing, talking loudly, coughing, and sneezing.^2,3^ In particular, coughing and sneezing are spasmodic events and multiphase dynamical phenomena. Hence, understanding its dynamics is non-trivial due to the interplay and competition of different forces.^4^ While expelling the respiratory liquid, the bulk fluid is converted into several polydisperse droplets^5^ via multiple intermediate processes. The occurrence of these events is analogous to that of liquid atomization processes.^6^

Talking loudly,^7^ coughing, and sneezing,^8^ cause a significant increase in pressure in the nasal cavity that tends to expel a cloud of respiratory liquid to the ambient air from the mouth within a microsecond. This dynamic event is accompanied by the combined occurrence of Kelvin–Helmholtz (K–H) and Rayleigh–Taylor (R–T) instabilities due to the generation of axial relative velocity and an acceleration field, respectively. Thus, the expelled respiratory liquid, in particular, coughing and sneezing, is a case where both the instabilities could occur. Therefore, it presents itself as a prominent example of the classical primary liquid atomization problem.^9^ The expelled respiratory liquid cloud is further ruptured into ligaments and drops, signifying the secondary atomization. The droplets generated out of the expelled respiratory liquid process are polydisperse in nature.^10^ These droplet ranges from sub-micron to hundreds of micrometers are the primary factor in airborne disease transmission. These respiratory droplets can act as a carrier of pathogens and allow them to transport in the air medium. The pathogens can stay alive inside the droplets, suspend in the ambient air for a long time, and spread the infections via this route.

Despite several studies that have attempted to uncover the dynamics involved in creating expelled respiratory droplets, little attention has been devoted to understanding the instabilities associated with these events, which are multiphysics in nature. Recent effort includes a realistic modeling of the characteristics of droplets originated from a human sneeze^11^ and their control strategies,^12,13^ survival of pathogens in the droplets deposited on surfaces,^14^ dispersion mechanism,^15^ and the effect of the respiratory droplets under different ambient conditions.^16^ However, the mechanism by which the droplets are formed close to the mouth has not been studied. Hence, there is a rising concern among researchers to unravel the entire dynamics with the aid of fluid mechanics tools, especially high-speed imaging.^17,18^ Lately, high-speed imaging has contributed to extending the knowledge on many scientific questions by revealing the intermediate events that happen during such spasmodic events. Therefore, the present work proposes an experiment with a soap film and air to visualize the dynamics of the expelled respiratory liquid sputum from a human and the associated mechanisms that govern the breakup process. From this standpoint, the breakup instability in the soap film serves as the simplified model of the respiratory liquid sheet to understand its dynamics. A simple and controlled experiment with the soap solution and air, although it is not performed on the exact geometry, captures the intermediate physical events that would occur during the destabilization of the respiratory liquid. This understanding is essential to elucidate the salient features of the respiratory expelled liquid and impervious airborne disease transmission.

**FIG. 1.**
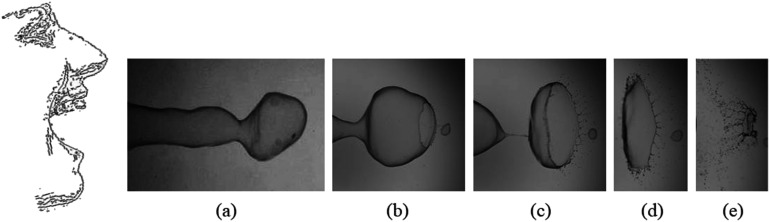
Illustration of instantaneous images of the expelled respiratory liquid and its breakup morphology. (a) When a sheet of sputum is expelled from a human, a liquid sheet gets destabilized to (b) form a drop (effect of the K–H instability). (c) The respiratory drop further gets “film ruptured” into ligaments (effect of the R–T instability). (d) The ligaments due to the effect of surface tension break up into (e) smaller drops (effect of the P–R instability). Note that these images (a)–(e) are obtained from a blown soap bubble with gravity pointing to the left and a mere representation of the intermediate events that occur while expelling the respiratory liquid. Likewise, the depicted images are highly exaggerated and not to the scale of a human mouth. The time interval between the two images is not equal.

During the process of expelling a respiratory liquid, a thin liquid jet or sheet of sputum is ejected out from the mouth to the ambient air resulting in ligaments. The ligaments will undergo a “film burst” process leading to the formation of a multitude of smaller fragments of varying sizes. The above process is very similar to the destabilization of the flapping liquid sheet, as illustrated in Fig. 1. The dynamics of a flapping sheet and its downstream processes have been extensively documented for the application of gas turbine atomizers.^19–21^ It should be mentioned that the breakup of thin liquid sheets^22^ is analogous to bursting the soap film.^23,24^ The expelled respiratory liquid as a ligament in the air stream will eventually burst^25^ or break up^26^ into smaller fragments. In the present work, an idealized system, including all these events in a single system, is investigated. In particular, the intermediate processes that occur between the formation of a thin liquid sheet and the bubbles fragmenting into smaller drops are of current interest.

The events mentioned above, in general, occur either independently or sequentially in different systems. The one striking feature from the present study is to show all these existing events during the process of expelling a respiratory liquid with a model system and characterize them for limited flow conditions.

We propose a simplified experimental model to investigate the intermediate physical events associated with expelling the respiratory liquid via flapping liquid sheets—these events resulting in numerous smaller fragments. We study the underlying dynamics using a model of the soap film while the air impinges on it. The velocity gradients between the air and the soap film initiate the K–H instability and make the sheet flap. Eventually, flapping induces the R–T instability that initiates the breakup. We hope that this study helps understand the ubiquitous phenomenon in a better way. This paper reports high-speed imaging in a simple laboratory experimental setup aimed at delineating the different mechanisms and quantifying their effects.

This paper is streamlined as follows: In Sec. II, the experimental setup used for this study is presented. The behavior of the flow system and scaling are presented in Secs. III and IV, respectively. We discussed the limitations of our predictions in Sec. V and summarized in Sec. VI.

## II. EXPERIMENTAL SETUP—CONCEPT AND DETAILS

**FIG. 2.**
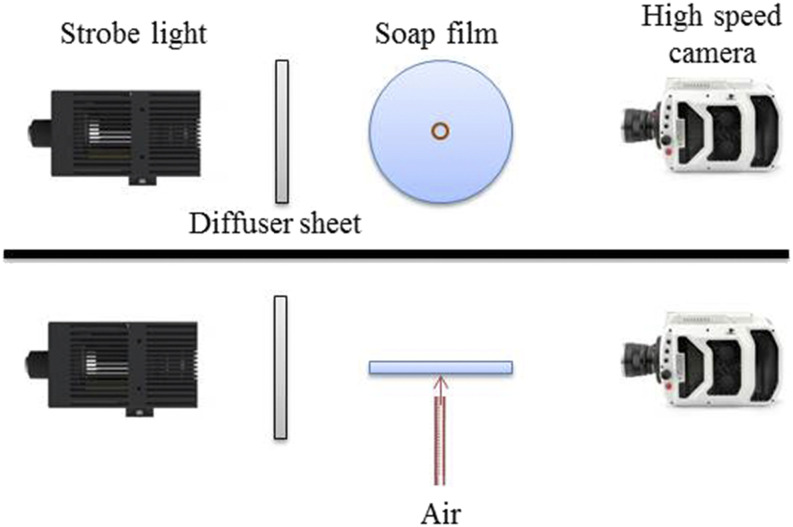
Sketch of the experimental setup.

A test rig capable of generating a cylindrical column of the bubble was designed and assembled. The rig consists of a circular frame and an air supply unit. A schematic of the experimental setup, along with a high-speed imaging setup, is shown in Fig. 2. The soap solution was prepared using 5% of liquid (Ivory) washing solution and 95% of distilled water by volume. The solution was well maintained below the critical micellar concentration. The same solution concentration was used for all the experiments. The surface tension of the solution was measured using the pendant drop method and a tensiometer test. The surface tension of the solution is ≈0.025 N/m. The other properties of the solution, such as density and kinematic viscosity, are assumed to be 1.23 kg/m^3^ and 10^−6^ m^2^/s, respectively.

A circular frame with a diameter of 200 mm was used to hold a soap solution as a thin film. The thin soap film was generated by immersing the circular frame into the soap solution and pulled out gently. The generated soap film was placed horizontally in the gravity field. We ensured that the soap film was stable, and there were no gradients in the radial direction. The entire experiments were conducted in the vapor saturated environment to avoid liquid evaporation.

A plane orifice nozzle with an exit diameter of 8 mm was used to create a high-velocity air jet. The nozzle was located 10 mm below the circular frame. The high-pressure air cylinder was used to supply the air to the nozzle. A high precision pressure regulator controls the airflow, and the flow rate was maintained. The jet velocity was measured using a pitot tube with an estimated accuracy of 2%. A steady flow of the air jet is placed below the circular frame to impinge high-velocity air perpendicular to the center of the thin film in the upward direction against gravity to produce a cylindrical column of the bubble.

To study the breakup behavior of the bubble column, a high-speed shadowgraphy technique was used. A bright field was created by illuminating the background with a high power LED light source. The diffuser sheet placed between the light source and the object helps produce the uniform light distribution. A Photron FastCam^^TM^^ high-speed imaging camera was used to capture the instantaneous images in shadowgraphy mode. To avoid streaks in the images, the light source was synchronized with the high-speed camera. The entire breakup events of the bubble column were captured at 10 000 f.p.s with an exposure time of 1/20 000 s, and its image resolution is 512 × 768 pixels. To capture the number of ligaments generated from the bubble burst, we captured the magnified events at 3600 f.p.s with an exposure time of 1/50 000 s, and its image resolution is 1024 × 1024 pixels. Special care was taken to enhance the reproducibility and quality of the experiments. Throughout the experiments, it was made sure that the soap film was steady and had a constant thickness initially.

## III. OBSERVATIONS—CHRONOLOGY OF THE FLOW SYSTEM

**FIG. 3.**
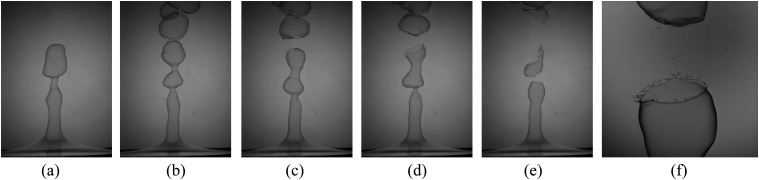
Typical time sequence of a thin soap film fragmentation process. (a) Image of the flapping sheet due to the impingement of air, (b) continuous formation of bubbles, (c) continuous decrease in the thickness that initiates a hole formation at a point and leads to (d) the rupture of the liquid sheet, (e) multiple bursts, and (f) the formation of ligaments. The bubbles were pinched off from the flapping sheet by their weight. In this sequence, the time interval between two consecutive frames is not the same.

We performed the experiments with the setup discussed in Sec. II, and the chronology of the expelling respiratory liquid is described here. The sequential behavior can be seen from Fig. 3 and is as follows: (a) flapping liquid sheet, (b) continuous formation of bubbles, (c) hole formation at a point, (d) rupture of the liquid sheet, (e) multiple bursts, and (f) the formation of ligaments. The bubbles pinch off from the flapping sheet by their own weight, as shown in Fig. 3(b). The rupturing and bursting process can be noted from Figs. 3(c)–3(e). The ligament formation is shown in Fig. 3(f). Note that, in this sequence shown in Fig. 3, the time interval between two consecutive frames is not equal. The present study is an attempt to characterize each event in detail.

We will classify the behavior of the flow system into four major events, as observed in the destabilization process of the cylindrical liquid sheet. The liquid sheet undergoes different stages such as (A) flapping, (B) rupture, (C) ligament, and (D) droplet formation before it forms as a stable droplet. The processes that lead to the sequential events will be discussed here with a few physical arguments.

In order to create a gas centered co-annular (cylindrical) liquid sheet, the air jet is allowed to impinge on the soap film. On a continuous supply of the air at the bottom surface of the soap film, the soap film expands both in axial and radial directions. Due to the airflow, the bubble expands in the axial direction. The interfacial area of the soap film increases, and the expansion is opposed by the surface tension force acting on the interface. In contrast, the expansion in the radial direction is limited by the frame edge. Note that the diameter of the bubble column is controlled by the diameter of the gas (air) jet.

The continuous domination of the airflow assists the cylindrical liquid (bubble) column to grow in the axial direction, as shown in Fig. 3(a). Due to the continuous co-flowing air stream, the stable bubble column will tend to destabilize by generating waves on its surface. The waves move toward the direction of the airflow, thus resulting in flapping. The flapping is due to the velocity difference between the interfaces.

The next event is addressed as rupturing the soap film, and it can be summarized as follows: Due to gravity or capillary suction, the liquid flows down in the bubble cap, leading to a thinner film. A hole eventually nucleates at any point in the film, and the hole expands. However, the point where the rupture happens is beyond the scope of the present study.

The corresponding event is bursting. This bursting is observed in two cases: (i) the bubble which is pinched off from the liquid sheet ruptures and tends to burst and (ii) the continuous sheet attached with the circular frame bursts continuously until all the mass of the soap film ruptures. The former is similar to the drop breakup when it is suddenly exposed to the high stream of air, and the latter is analogous to the flapping liquid sheet resulting in the formation of ligaments that eventually break up into smaller droplets.

The last event is the origination of tiny drops from the ligaments. In Sec. IV, the above-discussed events will be presented in detail.

## IV. RESULTS

### A. Flapping and breakup length

In this section, we will discuss the flapping motion of the cylindrical liquid sheet, and it is shown in Fig. 3(a). The liquid sheet flapping is associated with a velocity difference across the thin sheet, and it is the manifestation of the K–H instability.^27^ A liquid sheet on the still surrounding air is balanced by surface tension and modulated by the Bernoulli effect.

Flapping motion causes the annular liquid sheet to expand and contract in symmetric mode. As the surface wave propagates, the amplitude of oscillation increases and results in liquid sheet necking. Air at low impact velocity on a soap film causes the spherical bubbles to pinch off from the annular liquid sheet continuously. Figures 3(b)–3(e) depict the various time instants at which bubbles pinch off from the liquid sheet. One of the most striking features visible from these images is the breakup length. Here, the breakup length is defined as the length of the intact liquid sheet. It is measured from the horizontal soap film to a point at which it pinches off from the liquid sheet.

**FIG. 4.**
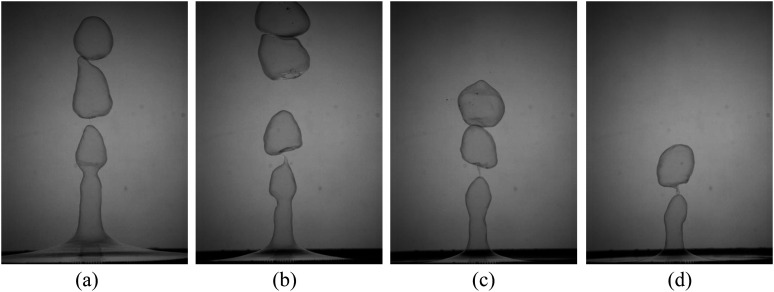
Breakup length for different flow conditions. (a) *We* = 79, (b) *We* = 177, (c) *We* = 315, and (d) *We* = 492. The length of the image is 3 cm. Note the number of bubbles in each image, and also, the breakup length decreases as airflow velocity increases.

Similar to Fig. 3, the breakup length was observed for other flow conditions as well, and it is shown in Fig. 4. Here, the images are presented for four different operating conditions. As can be noted, the breakup length decreases as the Weber number (velocity of the gas jet) increases. It can be observed that the diameter of the cylindrical sheet is approximately the same for all the conditions. This implies that the diameter of the cylindrical sheet depends only on the diameter of the air nozzle exit orifice.

The other factor is that the number of bubbles pinching off from the liquid sheet decreases as velocity increases. However, the numbers are not consistent, and it cannot be quantified. In other words, it can be observed while performing experiments that for the case of low Weber numbers, the number of bubbles pinching off is more, whereas for the high velocity, the number of bubbles pinching off is less or sometimes, the air jet ruptures even before the formation of bubbles. As can be noted from Fig. 3, the average number of bubbles formed are 4, 3, 2, and 1 for *We* = 79, *We* = 177, *We* = 315, and *We* = 492, respectively. In this study, the Weber number is defined by, $We=\frac{\rho_{g}V_{g}^{2}R}{\sigma }$, where *ρ*_*g*_ and *V*_*g*_ denote the density and velocity of the gas (air) jet, *R* denotes the radius of the gas jet, and *σ* denotes the surface tension.

**FIG. 5.**
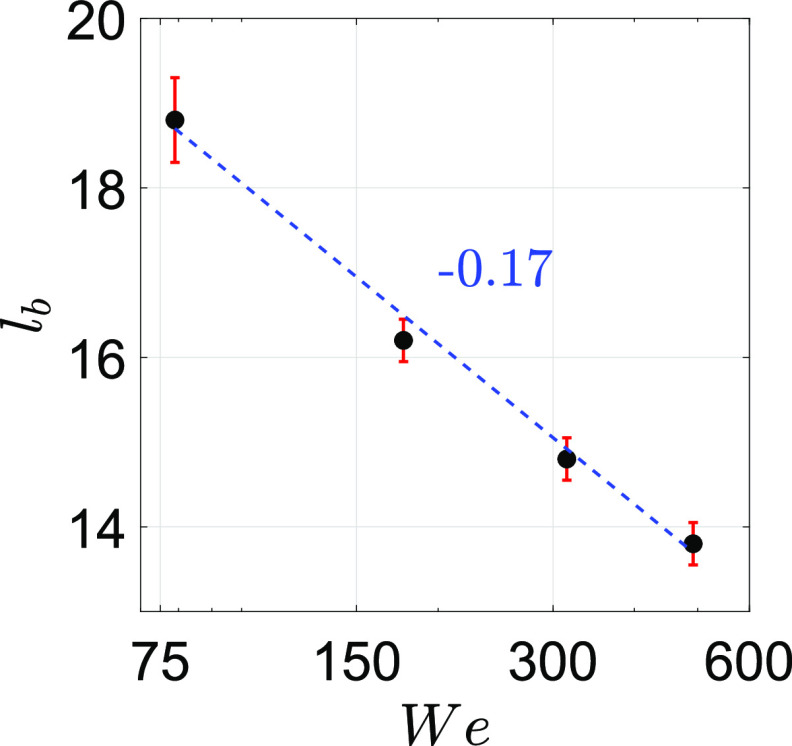
Influence of the K–H instability. Dimensionless breakup length (*l*_*b*_) as a function of Weber number (*We*). As can be seen, *l*_*b*_ ∼ *We*^−0.17^. Here, $We=\frac{\rho_{g}V_{g}^{2}R}{\sigma }$.

Figure 5 depicts the dimensionless breakup length (*l*_*b*_) as a function of Weber number. Here, *l*_*b*_ is defined by the dimensional breakup length ($l_{b}^{\prime }$) normalized by the radius of the gas jet (R), indicating $l_{b}=\frac{l_{b}^{\prime }}{R}$. The plot also shows that *l*_*b*_ decreases with an increase in *We*. It is due to the fact that inertial forces dominating the surface tension forces result in a decrease in *l*_*b*_ while increasing *We*. Note that *l*_*b*_ scales as *We*^−0.17^. The mean wavelength of the oscillations is observed to be the same for a range of *We*, henceforth, resulting in bubble formation of similar sizes. The size of the bubble is found to be around 20 mm from the images. It indicates that the increase in *We* (velocity of the gas jet) influences *l*_*b*_, and the pinched-off bubble size remains the same.^28^

### B. Rupture

**FIG. 6.**
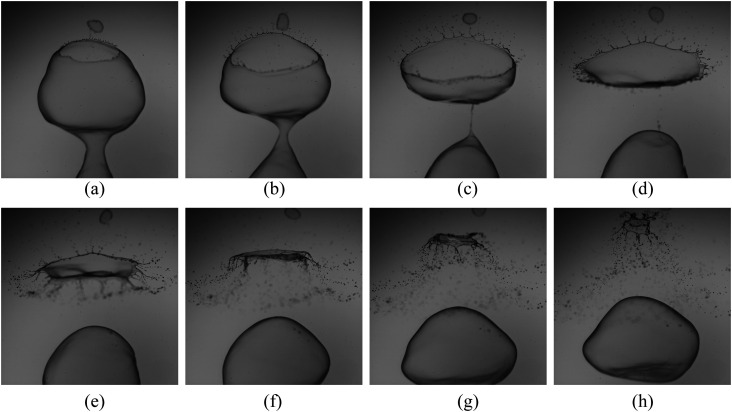
The sequence of high-speed images shows the complete evolution of bubble rupture morphology and pinching off from the cylindrical sheet at *We* = 492. (a) Initiation of rupture, (b) thin smooth rim transforming into a thicker corrugated rim, (c) rim breaking and formation of ligaments as well as ejection of film drops, (d) change of orientation, (e) inflation of the bubble into a pancake or jellyfish shape, (f) folding the sheet in the reversal direction, (g) collapsing the bubble, and (h) faster ejection of drops through ligaments. The length of the image is 20 mm.

The complete sequential behavior of rupture on a cylindrical liquid sheet is shown in Fig. 6. These instantaneous images were captured at 3600 f.p.s with an exposure time of 500 *µ*s. The inception of rupture occurs at a point in the liquid sheet where the sheet thickness tends to zero. In other words, the thin film’s initial puncture is localized preferentially at the point where the normal stresses are large. The rupture also occurs individually on each bubble disintegrated from the liquid sheet. The bubble diameter is equivalent to the wavelength of the liquid sheet as a result of K–H instability. The rupture event continues until the whole liquid sheet deforms.

On continuous impingement of air, the soap film ruptures at some critical point without any external disturbances by forming a small hole (internal pressure will be enough to break the bubble, causing it to burst). It is worth mentioning that the rupture may happen at multiple points simultaneously, leading to multiple bursts. Note that the images were captured using a high-speed camera representing two-dimensional projections of the three-dimensional motion; only bubbles with axisymmetric oscillations were selected for the data analysis. The rupture that can be visualized from the image was only considered and reported.

First, the bubble is in the form of a spherical shape and would like to retain its shape. The puncture or rupture happens from the upstream or downstream. It is common that whenever a bubble ruptures, the droplet or fragments are formed by the retraction of the thin film, as shown in Fig. 6. The sheet retraction caused by the rupture differs depending upon the velocity conditions. Followed by these events, there will be a formation of ligaments, leading to the formation of other sets of droplets [Figs. 6(b)–6(d)]. A similar thing was observed while the bubble tends to burst. During the course, the retracting sheet flattens the bubble-like pancake shape and then spirals up to shatter the liquid sheet into smaller fragments,^25,26,29,30^ as shown in Figs. 6(e)–6(h).

### C. Ligament formation

**FIG. 7.**
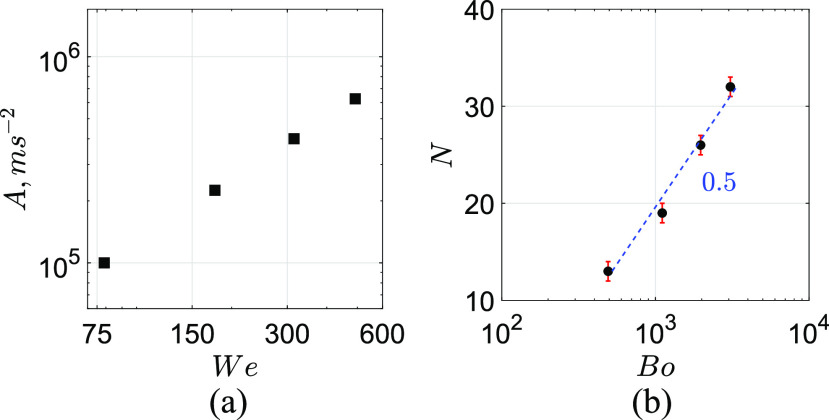
Influence of the R–T instability. (a) Acceleration of the thin liquid sheet (*A*) in m s^−2^ as a function of Weber number (*We*). (b) Number of ligaments formed (*N*) as a function of Bond number (*Bo*). Here, $Bo=\frac{\rho_{g}R_{b}^{2}A}{\sigma }$. (b) signifies $N∼\sqrt{Bo}$.

The event of rupturing or bursting a spherical bubble leads to the formation of ligaments. The number of ligaments depends upon the acceleration experienced by the spherical bubble while rupturing. Here, acceleration (*A*) is a dimensional quantity obtained by $A=\frac{V_{g}^{2}}{R}$ and Bond number, $Bo=\frac{\rho_{g}R_{b}^{2}A}{\sigma }$. In addition, *R*_*b*_ represents the radius of the bubble. Figure 7(a) shows the acceleration (*A*) as a function of Bond (*Bo*) number. As can be seen, as *We* increases, *A* tends to increase. Both *A* and *We* increase because the gas (air) velocity increases. R–T instability occurs in a spherical bubble^31^ that leads to the formation of ligaments in several instances, which includes breakup either due to viscous drainage^32^ or destabilized by a laser source^33^ or by a combustion source.^34^ We confirmed and stated that it yields the same number of ligaments irrespective of their sources. With this, we also characterize the number of formation of ligaments (*N*) for the wide range of Bond numbers (*Bo*), as shown in Fig. 7(b). As can be seen, increasing the value of *Bo* tends to increase *N*. It is due to the dominance of inertial forces driven by acceleration over the surface tension forces. Note that the trend observed for a cylindrical thin liquid sheet ($N∼\sqrt{Bo}$) is consistent with the aforementioned earlier studies.^31–36^

### D. Drop formation

**FIG. 8.**
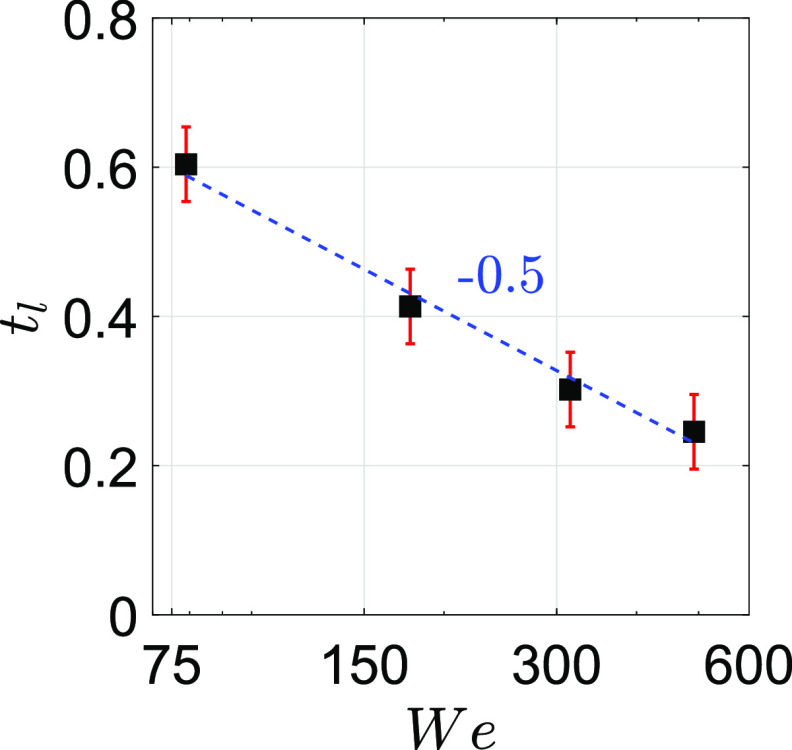
Thickness of the ligament (*t*_*l*_) as a function of Weber number (*We*). Here, $We=\frac{\rho_{g}V_{g}^{2}R}{\sigma }$. Each ligament breaks into tiny drops due to the influence of the P–R instability. Note that *t*_*l*_ ∼ *We*^−0.5^.

It is well known that each ligament will tend to break up further into drops. It is due to the Plateau–Rayleigh (P–R) instability. Here, we discuss the thickness (*t*_*l*_) of each ligament that is formed. Figure 8 shows the thickness of the ligament (*t*_*l*_) as a function of Weber number (*We*). Here, *t*_*l*_ is normalized as $t_{l}=\frac{\pi D_{b}}{NR}$, where *D*_*b*_ refers to the diameter of the bubble before rupturing. Note that each ligament thickness reduces due to an increase in the number of ligaments. An increase in *We* of the flow increases the number of ligaments formed (*N*), resulting in thinner ligaments. In other words, increasing *We* leads to thinner ligaments. Therefore, the trend of *t*_*l*_ decreases as an increase in *We* is quite a common factor. Note that *t*_*l*_ scales as *We*^−0.5^. Later, each ligament will end up in smaller drops due to the P–R instability.

## V. DISCUSSION

In the present work, we have delineated the instabilities associated with expelling the respiratory liquid by a human by the idealization of destabilizing a thin liquid sheet. We show that the event is governed by three instability mechanisms, namely, Kelvin–Helmholtz instability, Rayleigh–Taylor instability, and Plateau–Rayleigh instability. With careful experiments, we also confirm that the K–H instability only provides a mechanism for the thin sheet to flap and form as a rim at the edge, while rupturing the rim is by thinning a liquid sheet, followed by destabilization of the liquid sheet edge, which involves both R–T and P–R instability mechanisms. The present work indicates that the axially long cylindrical liquid sheet of 20 mm of diameter with a thickness of 1 mm would break into a 10 mm bubble (drop) due to the K–H instability. The bubble further ruptures into a 1 mm thickness of the ligament due to the R–T instability. This ligament tends to tiny drops in the order of 10 *μ*m obeying the P–R instability. It is to be noted that our experiments are within a low viscous regime, where surface tension dominates any viscous effects. In addition, it provides the platform to showcase the influence of K–H and R–T instabilities with surface tension in the absence of viscous effects. Furthermore, the dynamics of these drops and their transmission routes could be altered depending upon the conditions such as wind speed, temperature, and humidity.^37–39^ This study also complements the work of blowing the soap film^40^ as well as other exhaustive experiments on coaxial atomizers.^28,32,41,42^

The experimental setup considered in the present study gives glimpses into the dynamics of sneezing and lends itself well for quantitative analysis. Although this has widened the understanding of sneezing for the limited flow conditions, we are aware that the present study also proposes more exciting questions. It is worthwhile to mention that comparisons with the existing literature for sneezing could not be performed because the parameters that govern the event are rather different. The complete morphology of sneezing phenomena can be understood only by conducting experiments with a fluid with similar properties as saliva and mucus, which generally exhibit non-Newtonian behavior. In general, the typical velocity of the cough or sneeze is not well documented, even for a healthy person. To date, such data for an infected person are still in the scientific perusal stage.

On the other hand, there are enough possibilities by which one can generate a droplet cloud while talking loudly or singing. In such instances, the dynamics could be entirely different because multiple ejections happen at very low velocities. These complexities suggest that current researchers include multiple hydrodynamic instabilities and develop a holistic combined model. For example, if aerosol generation by sneezing is the outcome of the simultaneous effect of both K–H and R–T instabilities, a flow system that mimics both mechanisms is cumbersome and even harder to perform *in situ* measurements. However, a detailed comparison between the theoretical predictions^35,36,43^ of both mechanisms and the observations from the real condition experiments deserves an independent study in the near future.

## VI. SUMMARY

The present study highlights the role of hydrodynamic instabilities in atomizing expelled respiratory human liquids. To achieve this purpose, we have idealized a sneeze or any other expelling respiratory event by a flow system comprising a soap film and an air jet. We have identified the various intermediate events encountered by the annular liquid sheet to form stable droplets. We observed various hydrodynamic instabilities that are responsible for each event with the aid of high-speed imaging. We observed that the cylindrical liquid sheet undergoes flapping, rupture, ligament, and droplet formation. We quantified the dynamics of a thin annular liquid sheet for a range of Weber and Bond numbers. In this context, we showed that the dimensionless breakup length of the expelled respiratory liquid film scales as a function of Weber number, *l*_*b*_ ∼ *We*^−0.17^, while the number of ligaments scales as a function of Bond number, $N∼\sqrt{Bo}$. In addition, we also showed that the thickness of the ligament (*t*_*l*_) is a strong function of the Weber number. Finally, we hope that the present study would help the current researchers gain momentum to explore hydrodynamic instabilities during sneezing. This understanding is crucial to elucidate the aerosol formation and to mitigate disease transmission through expelled respiratory droplets.

## DATA AVAILABILITY

The data that support the findings of this study are available from the corresponding author upon reasonable request.
